# Respiratory Rate Variability as a Prognostic Factor in Hospitalized Patients Transferred to the Intensive Care Unit

**DOI:** 10.7759/cureus.2100

**Published:** 2018-01-23

**Authors:** Daniel Garrido, Justin J Assioun, Anahit Keshishyan, Marcos A Sanchez-Gonzalez, Bishoy Goubran

**Affiliations:** 1 Department of Research, FIU; 2 Department of Research, St George's University; 3 Department of Research, Larkin Community Hospital; 4 Division of Clinical and Translational Research, Larkin Community Hospital

**Keywords:** respiratory rate variability, prognosis, icu, icu admission

## Abstract

Introduction

Increasing mortality rates within the intensive care unit (ICU) is an ever growing problem, ultimately leading to increases in the cost of healthcare expenditures. Currently, there are attempts to use guidelines in the hospital setting to predict overall mortality in critically ill patients. However, a predictor of subsequent ICU admissions remains to be explored. Recent data has shown the importance of monitoring respiratory rate variability (RRV) as a useful predictor of the deterioration of patients. Respiratory rate, in comparison to blood pressure or pulse rate, is deemed as the better determinant in identifying high-risk patients.

Aim

Our study aims to assess the role of RRV monitoring as a potential prognostic marker predictive of ICU admission.

Results

There was a significant (p = 0.009) increase in RRV between the third and fourth set of respiratory rates prior to ICU admission, such that coefficient of variation percentage (CV%) increased from 0.3% (95% confidence interval (CI): 0.09 - 0.42) to 0.7% (95% CI: 0.04 - 0.9) about 12 hours before admission to the ICU independent from diagnosis.

Conclusion

Using elevated RRV as a signal may be a useful prognostic tool in providing early intervention, thus reducing the incidence of subsequent morbidity and mortality in patients that might necessitate an ICU admission.

## Introduction

Patient mortality within the intensive care unit (ICU) setting is an ever-growing problem affecting the healthcare system. Increased ICU admissions and mortality rates lead to increased healthcare expenditures [1-2]. With these concerns, multiple models have been created to stratify patients within this setting and predict overall prognosis, such as Quick Sepsis-related Organ Failure Assessment (qSOFA) and Systemic Inflammatory Response Syndrome (SIRS). Although both qSOFA and SIRS have been widely used as prognostic indicators specifically for patients with sepsis, there have been some questions about their limitations as a global prognostic indicator [3]. Moreover, a simple indicator of deterioration that may predict a subsequent ICU admission has yet to be explored.

Normal breathing exhibits a relatively constant rate and tidal volume that together constitute normal respiratory rhythm. However, variations within this respiratory rhythm are labeled as respiratory rate variability (RRV) [4]. Changes in respiratory rate (RR) is an important indicator which often precedes major clinical manifestation of serious complications, such as respiratory tract infections, respiratory depression, and failure [5]. In contrast with pulse rate, respiratory rate is a better predictor of stability of patients [6]. High respiratory rates have been shown to predict the majority of in-hospital cardiac arrest and admission to intensive care [7]. Alterations in RRV are prominently seen in patients with chronic comorbidities, such as heart failure and chronic obstructive pulmonary disease (COPD) [8]. However, increased RRV is also observed in patients during periods of critical illness, specifically in patients with heart failure immediately prior to decompensation [8]. RRV measure has been utilized within the ICU setting where continuous monitoring of RRV demonstrated that an alteration in RRV was more likely associated with extubation failure [9]. Identifying the deteriorating status of a patient in a timely manner is critical in warranting prompt intervention and prevention of poor clinical outcomes [10]. It is, therefore, the aim of this study to assess the role of RRV as a global prognostic marker for ICU admission in patients transferred from the medical-surgical floor. We hypothesize that alterations in RRV would be associated with ICU admissions.

## Materials and methods

In this observational retrospective chart review, we analyzed a total of 50 (females = 28) consecutive patients within the inpatient setting for at least 48 hours prior to being transferred to the ICU. A patient transferred from the regular internal medicine floor to the ICU setting was considered as an indicator of a worsening prognosis. Inclusion criteria for this study were admittance to the inpatient setting for 48 hours before admission to the ICU, as well as eight consecutive sets of vital signs taken every six hours (each) during the 48 hour period. Trauma, post-surgical, and cancer patients were excluded from the study. Subsequent pairs of respiratory rates measurements were paired to calculate the coefficient of variation percentage (CV%) as a surrogate of RRV. The four pairs of RRV were then analyzed for changes in CV% over time by means of analysis of variance (ANOVA) with repeated measures to establish if there was any statistically significant change.

## Results

**Figure 1:**
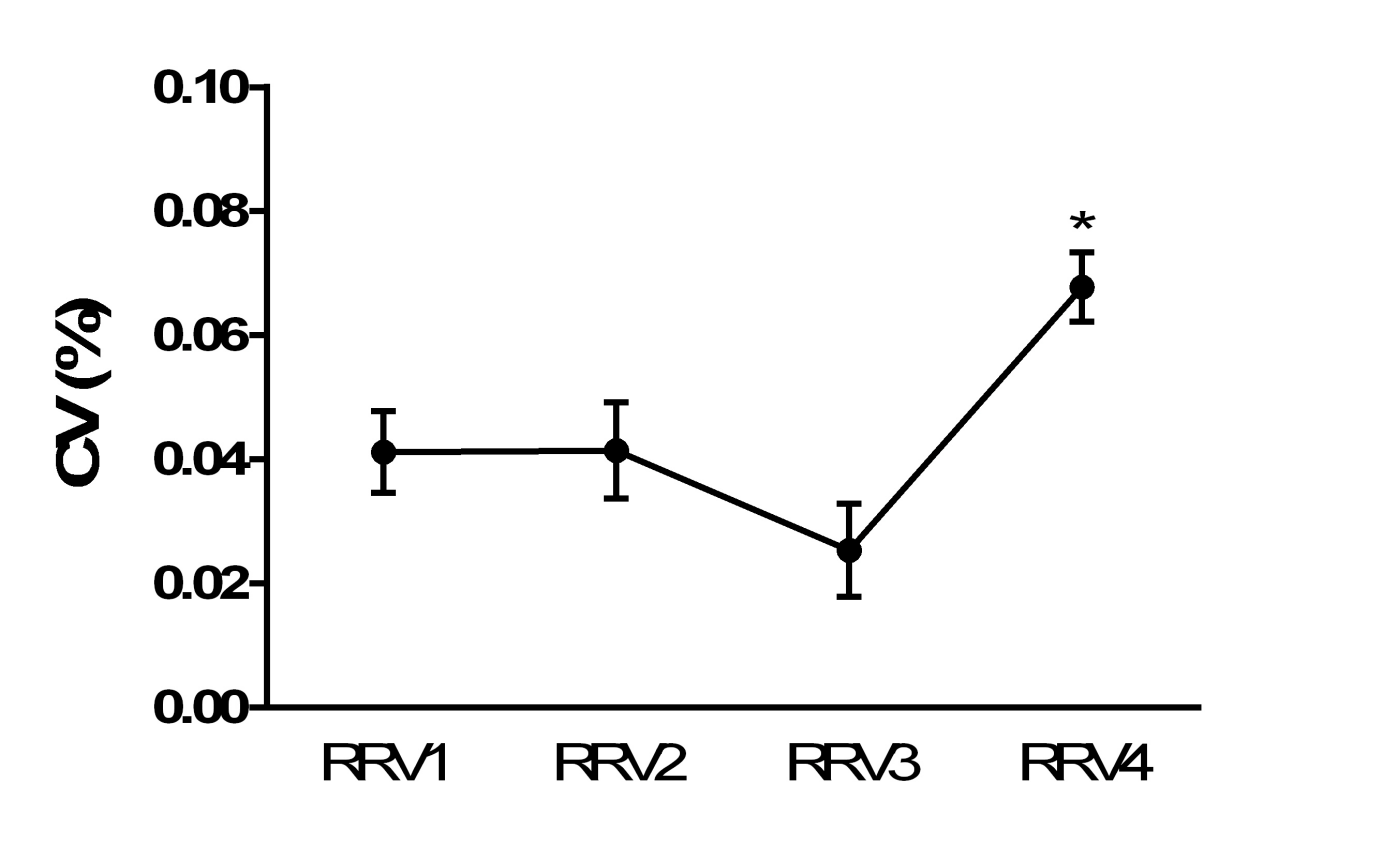
Changes of respiratory rate variability before intensive care unit admission * p < .001 CV%: coefficient of variation percentage; RRV: respiratory rate variability.

Subject characteristics were age (58.1 ± 7.1 years) (mean ± standard deviation (M ± SD)) and body mass index (BMI) (27.6 ± 12.2 kg/m2). There was a significant (p = 0.009) increase in RRV between the third and fourth set of respiratory rates prior to ICU admission, such that CV% increased from 0.3% (95% confidence interval (CI): 0.09 - 0.42) to 0.7% (95% CI: 0.04 - 0.9) about 12 hours before admission to the ICU independent from diagnosis as displayed in Figure 1. We did not find any significant difference between genders. 

## Discussion

This study revealed that alterations in RRV are associated with admission to the ICU. Data have elucidated a statistically significant increase in RRV between the third and fourth set of respiratory rates prior to the ICU admission. We believe this might be a prognostic pattern predictive of the deterioration of the patients in this population necessitating their admission to the ICU.

Vital signs indeed play an essential role in portraying an overall picture of a patient’s status. Monitoring for changes in a patient’s vital signs can help predict the onset of complications. Yet, when utilizing vitals as a predictor of worsening patient status, the question remains: Are all vitals created equal? In their study, Subbe, et al. deemed respiratory rate, in comparison to blood pressure or pulse rate, as the better determinant in identifying high-risk patient groups [11]. Thus, an elevated RR is a strong and specific predictor of serious adverse events.

The use of RRV has been shown to identify breath-to-breath variations as a physiological response to respiratory output [12]. An alteration in RRV has been described with increased hypercapnia and dyspnea, indicating a decreased adaptive response to tolerate an increased workload [13]. Furthermore, studies have shown that the applicability of RRV has even extended to the ICU setting as an early warning clinical marker and predictive tool for the identification of extubation failure [9, 14]. Interestingly, data have shown that the occurrence of one or more respiratory rates exceeding 27 breaths per minute over a 72-hour period had a high specificity (0.83) in predicting cardiopulmonary arrest [15]. Taken together, these studies suggest that elevated RRVs may be useful as a surrogate marker for acute patient deterioration.

Ventilation is a controlled modality with an array of responses enabling adequate gas exchange, thus adjusting the levels of oxygen (PaO2), carbon dioxide (PaCO2), and potential of hydrogen (pH) within a normal physiological range. The respiratory rate and tidal volume are modulated by higher cortical centers, such as the medullary respiratory center, which is responding to subtle changes in arterial blood gas parameters [7]. Pathological conditions altering PaO2, PaCO2, or pH changes input from both the carotid body and the medullary chemoreceptors, affecting both the rate and tidal volume [16]. Hence, the respiratory rate is responsive to subtle pathological changes and may, therefore, serve as an early sign of deterioration and underlying disease manifesting as changes in RRV, thus predicting a transfer to the ICU.

Indubitably, admission to the ICU is associated with increased rates of morbidity and mortality. We, therefore, suggest that increased changes in the RRV are valuable prognostic predictors that a patient’s health status is deteriorating and thus necessitates a transfer to the ICU. By monitoring the RRV, physicians can have an early indicator of a deterioration of the patient and thus promptly intervene prior to patient desaturation necessitating an urgent transfer. The strengths of this study include the presence of multiple sets of respiratory rates utilizing a within-subject approach to minimize confounding bias. However, there were several limitations, which include a small sample size and a population consisting of only internal medicine floor patients. The study was unable to control for the different prognostic factors for each patient diagnosis, possibly introducing bias, as this was an observational study and there was no control group.

## Conclusions

These data suggest that an increase in RRV could be indicative of a worsening prognosis in patients within the inpatient setting. Thus, we believe RRV can be used as an early marker of potential health complications. However, the association of RRV changes and worsening prognosis, regardless of the diagnosis, may provide an avenue to investigate the role of RRV in specific patient diagnoses. Prospective studies aimed at understanding the clinical value of RRV in the hospital setting are warranted.
